# 2-Amino-5-bromo­pyridinium 5-chloro-2-hy­droxy­benzoate

**DOI:** 10.1107/S160053681300665X

**Published:** 2013-03-16

**Authors:** Kaliyaperumal Thanigaimani, Nuridayanti Che Khalib, Suhana Arshad, Ibrahim Abdul Razak

**Affiliations:** a School of Physics, Universiti Sains Malaysia, 11800 USM, Penang, Malaysia

## Abstract

In the 5-chloro­salicylate anion of the title salt, C_5_H_6_BrN_2_^+^·C_7_H_4_ClO_3_^−^, an intra­molecular O—H⋯O hydrogen bond with an *S*(6) graph-set motif is formed, so that the anion is essentially planar with a dihedral angle of 1.3 (5)° between the benzene ring and the carboxyl­ate group. In the crystal, the protonated N atom and the 2-amino group of the cation are hydrogen bonded to the carboxyl­ate O atoms *via* a pair of N—H⋯O hydrogen bonds, forming an *R*_2_^2^(8) ring motif. The crystal structure also features N—H⋯O and weak C—H⋯O inter­actions, resulting in a layer parallel to the (10-1) plane.

## Related literature

For background to the chemistry of substituted pyridines, see: Pozharski *et al.* (1997 ▶); Katritzky *et al.* (1996 ▶). For related structures, see: Goubitz *et al.* (2001 ▶); Quah *et al.* (2010 ▶); Thanigaimani *et al.* (2013 ▶); Raza *et al.* (2010 ▶). For hydrogen-bond motifs, see: Bernstein *et al.* (1995 ▶). For bond-length data, see: Allen *et al.* (1987 ▶). For stability of the temperature controller used for data collection, see: Cosier & Glazer (1986 ▶). 
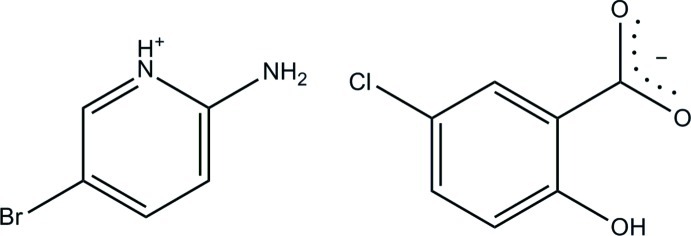


## Experimental

### 

#### Crystal data

C_5_H_6_BrN_2_^+^·C_7_H_4_ClO_3_^−^
*M* *_r_* = 345.58
Monoclinic, 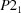

*a* = 8.9769 (17) Å
*b* = 5.6601 (12) Å
*c* = 12.753 (2) Å
β = 90.662 (5)°
*V* = 647.9 (2) Å^3^
*Z* = 2
Mo *K*α radiation
μ = 3.38 mm^−1^
*T* = 100 K
0.31 × 0.04 × 0.03 mm

#### Data collection

Bruker SMART APEXII DUO CCD area-detector diffractometer
Absorption correction: multi-scan (*SADABS*; Bruker, 2009 ▶) *T*_min_ = 0.417, *T*_max_ = 0.894
8030 measured reflections
4233 independent reflections
3014 reflections with *I* > 2σ(*I*)
*R*_int_ = 0.087

#### Refinement

*R*[*F* ^2^ > 2σ(*F* ^2^)] = 0.046
*wR*(*F* ^2^) = 0.091
*S* = 0.91
4233 reflections
188 parameters
1 restraint
H atoms treated by a mixture of independent and constrained refinement
Δρ_max_ = 0.84 e Å^−3^
Δρ_min_ = −0.98 e Å^−3^
Absolute structure: Flack (1983 ▶), 1558 Friedel pairs
Flack parameter: 0.037 (11)

### 

Data collection: *APEX2* (Bruker, 2009 ▶); cell refinement: *SAINT* (Bruker, 2009 ▶); data reduction: *SAINT* ; program(s) used to solve structure: *SHELXTL* (Sheldrick, 2008 ▶); program(s) used to refine structure: *SHELXTL*; molecular graphics: *SHELXTL*; software used to prepare material for publication: *SHELXTL* and *PLATON* (Spek, 2009 ▶).

## Supplementary Material

Crystal structure: contains datablock(s) global, I. DOI: 10.1107/S160053681300665X/is5251sup1.cif

Structure factors: contains datablock(s) I. DOI: 10.1107/S160053681300665X/is5251Isup2.hkl

Supplementary material file. DOI: 10.1107/S160053681300665X/is5251Isup3.cml

Additional supplementary materials:  crystallographic information; 3D view; checkCIF report

## Figures and Tables

**Table 1:** Hydrogen-bond geometry (Å, °)

*D*—H⋯*A*	*D*—H	H⋯*A*	*D*⋯*A*	*D*—H⋯*A*
O3—H1O3⋯O2	0.77 (8)	2.02 (5)	2.553 (4)	127 (6)
N1—H1N1⋯O2^i^	0.86 (5)	1.82 (5)	2.666 (4)	172 (4)
N2—H1N2⋯O1^i^	0.96 (6)	1.81 (6)	2.770 (5)	175 (4)
N2—H2N2⋯O1^ii^	0.88 (5)	1.95 (5)	2.799 (5)	164 (3)
C8—H8*A*⋯O3^iii^	0.95	2.53	3.410 (5)	154

Symmetry codes: (i) 
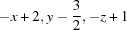
; (ii) 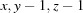; (iii) 
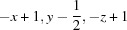
.

**Fig. 1.**
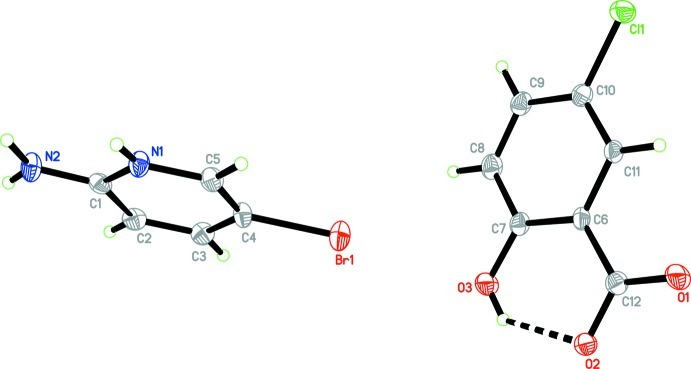
The molecular structure of the title compound with atom labels with 50% probability displacement ellipsoids.

**Fig. 2.**
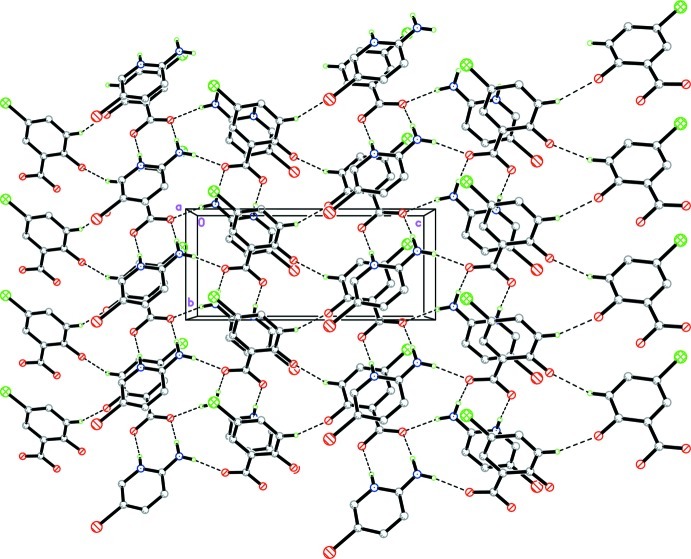
The crystal packing of the title compound. The H atoms not involved in the intermolecular interactions (dashed lines) have been omitted for clarity.

## supplementary crystallographic information

### Comment

Pyridine and its derivatives play an important role in heterocyclic chemistry (Pozharski *et al.*, 1997; Katritzky *et al.*, 1996). They are often involved in hydrogen-bonding interactions. Related crystal structures of 2-amino-5-bromopyridine (Goubitz *et al.*, 2001), 2-amino-5-bromopyridinium 2-hydroxybenzoate (Quah *et al.*, 2010) and 2-amino-5-methylpyridinium 2-hydroxy-5-chlorobenzoate (Thanigaimani *et al.*, 2013) have been reported. In order to study potential hydrogen-bonding interactions, the crystal structure determination of the title compound (I) was carried out.

The asymmetric unit (Fig. 1) contains one 2-amino-5-bromopyridinium cation and one 5-chlorosalicylate anion. An intramolecular O3–H1O3···O2 hydrogen bond in the 5-chlorosalicylate anion generates an S(6) ring motif (Bernstein *et al.*, 1995). This motif is also observed in the crystal structures of 5-chloro-2-hydroxybenzoic acid (Raza *et al.*, 2010). In the 2-amino-5-bromopyridinium cation, a wide angle [122.5 (4)°] is subtended at the protonated N1 atom. The 2-amino-5-bromopyridinium cation and 5-chlorosalicylate anion are essentially planar, with a maximum deviation of 0.008 (4) Å for atom N2 and 0.026 (4) Å for atom O1, respectively. The bond lengths (Allen *et al.*, 1987) and angles are normal.

In the crystal packing (Fig. 2), the protonated N1 atom and a nitrogen atom of the 2-amino group (N2) are hydrogen-bonded to the carboxylate oxygen atoms (O1 and O2) *via* a pair of intermolecular N1—H1N1···O2^i^ and N2—H1N2···O1^i^ hydrogen bonds (symmetry code in Table 1), forming a ring motif *R*_2_^2^(8) (Bernstein *et al.*, 1995). The crystal structure is further stabilized by N2—H2N2···O1^ii^ and C8—H8A···O3^iii^ (symmetry codes in Table 1) intermolecular interactions. These interactions have resulted in a molecular layer parallel to the (101) plane. This crystal structure is isomorphous to the crystal structure of 2-amino-5-methylpyridinium 2-hydroxy-5-chlorobenzoate (Thanigaimani *et al.*, 2013).

### Experimental

Hot methanol solutions (20 ml) of 2-amino-5-bromopyridine (43 mg, Aldrich) and 5-chlorosalicylic acid (43 mg, Aldrich) were mixed and warmed over a heating magnetic stirrer hotplate for a few minutes. The resulting solution was allowed to cool slowly at room temperature and crystals of the title compound (I) appeared after a few days.

### Refinement

O- and N-bound H atoms were located in a difference Fourier map and allowed to be refined freely [O—H = 0.77 (8) Å and N—H = 0.86 (5)–0.96 (6) Å]. The remaining H atoms were positioned geometrically (C—H = 0.95 Å) and refined using a riding model, with *U*_iso_(H) = 1.2*U*_eq_(C). Eight outliers were omitted (-4 -3 1, -1 -2 2, 1 0 5, 3 2 7, -1 -2 3, -1 0 5, 2 4 0, 2 -4 0) in the final refinement.

### Crystal data

**Table:**

C_5_H_6_BrN_2_^+^·C_7_H_4_ClO_3_^−^	*F*(000) = 344
*M**_r_* = 345.58	*D*_x_ = 1.771 Mg m^−^^3^
Monoclinic, *P*2_1_	Mo *K*α radiation, λ = 0.71073 Å
Hall symbol: P 2yb	Cell parameters from 1541 reflections
*a* = 8.9769 (17) Å	θ = 3.9–25.8°
*b* = 5.6601 (12) Å	µ = 3.38 mm^−^^1^
*c* = 12.753 (2) Å	*T* = 100 K
β = 90.662 (5)°	Needle, colourless
*V* = 647.9 (2) Å^3^	0.31 × 0.04 × 0.03 mm
*Z* = 2	

### Data collection

**Table:**

Bruker SMART APEXII DUO CCD area-detector diffractometer	4233 independent reflections
Radiation source: fine-focus sealed tube	3014 reflections with *I* > 2σ(*I*)
Graphite monochromator	*R*_int_ = 0.087
φ and ω scans	θ_max_ = 32.7°, θ_min_ = 2.3°
Absorption correction: multi-scan (*SADABS*; Bruker, 2009)	*h* = −13→13
*T*_min_ = 0.417, *T*_max_ = 0.894	*k* = −7→8
8030 measured reflections	*l* = −19→19

### Refinement

**Table:**

Refinement on *F*^2^	Secondary atom site location: difference Fourier map
Least-squares matrix: full	Hydrogen site location: inferred from neighbouring sites
*R*[*F*^2^ > 2σ(*F*^2^)] = 0.046	H atoms treated by a mixture of independent and constrained refinement
*wR*(*F*^2^) = 0.091	*w* = 1/[σ^2^(*F*_o_^2^) + (0.*P*)^2^] where *P* = (*F*_o_^2^ + 2*F*_c_^2^)/3
*S* = 0.91	(Δ/σ)_max_ < 0.001
4233 reflections	Δρ_max_ = 0.84 e Å^−^^3^
188 parameters	Δρ_min_ = −0.98 e Å^−^^3^
1 restraint	Absolute structure: Flack (1983), 1558 Friedel pairs
Primary atom site location: structure-invariant direct methods	Flack parameter: 0.037 (11)

### Special details

**Table:**

Experimental. The crystal was placed in the cold stream of an Oxford Cryosystems Cobra open-flow nitrogen cryostat (Cosier & Glazer, 1986) operating at 100.0 (1) K.
Geometry. All e.s.d.'s (except the e.s.d. in the dihedral angle between two l.s. planes) are estimated using the full covariance matrix. The cell e.s.d.'s are taken into account individually in the estimation of e.s.d.'s in distances, angles and torsion angles; correlations between e.s.d.'s in cell parameters are only used when they are defined by crystal symmetry. An approximate (isotropic) treatment of cell e.s.d.'s is used for estimating e.s.d.'s involving l.s. planes.
Refinement. Refinement of *F*^2^ against ALL reflections. The weighted *R*-factor *wR* and goodness of fit *S* are based on *F*^2^, conventional *R*-factors *R* are based on *F*, with *F* set to zero for negative *F*^2^. The threshold expression of *F*^2^ > σ(*F*^2^) is used only for calculating *R*-factors(gt) *etc*. and is not relevant to the choice of reflections for refinement. *R*-factors based on *F*^2^ are statistically about twice as large as those based on *F*, and *R*-factors based on ALL data will be even larger.

### Fractional atomic coordinates and isotropic or equivalent isotropic displacement parameters (Å^2^)

**Table:**

	*x*	*y*	*z*	*U*_iso_*/*U*_eq_	
Br1	0.79689 (4)	0.54521 (9)	0.42592 (3)	0.02463 (10)	
Cl1	0.50091 (11)	0.29983 (19)	0.90762 (8)	0.0253 (2)	
O1	0.8634 (3)	1.0293 (8)	0.87224 (17)	0.0248 (6)	
O2	0.8465 (3)	1.1615 (5)	0.7080 (2)	0.0205 (6)	
O3	0.6812 (3)	0.9496 (7)	0.5736 (2)	0.0239 (7)	
N1	0.9596 (4)	0.0044 (6)	0.2466 (2)	0.0190 (8)	
N2	0.9338 (4)	−0.1193 (7)	0.0762 (3)	0.0209 (8)	
C1	0.8982 (4)	0.0324 (11)	0.1506 (2)	0.0177 (7)	
C2	0.8005 (4)	0.2245 (7)	0.1337 (3)	0.0196 (8)	
H2A	0.7561	0.2495	0.0666	0.024*	
C3	0.7703 (4)	0.3746 (7)	0.2152 (3)	0.0196 (8)	
H3A	0.7039	0.5034	0.2050	0.024*	
C4	0.8375 (4)	0.3379 (7)	0.3134 (3)	0.0188 (8)	
C5	0.9301 (4)	0.1523 (7)	0.3275 (3)	0.0207 (8)	
H5A	0.9748	0.1252	0.3943	0.025*	
C6	0.7012 (4)	0.8297 (7)	0.7544 (3)	0.0162 (7)	
C7	0.6406 (4)	0.8057 (8)	0.6536 (3)	0.0177 (7)	
C8	0.5392 (4)	0.6258 (7)	0.6314 (3)	0.0223 (9)	
H8A	0.4986	0.6110	0.5626	0.027*	
C9	0.4971 (4)	0.4687 (7)	0.7085 (3)	0.0210 (8)	
H9A	0.4290	0.3447	0.6932	0.025*	
C10	0.5563 (4)	0.4956 (7)	0.8089 (3)	0.0187 (9)	
C11	0.6566 (4)	0.6721 (7)	0.8323 (3)	0.0169 (8)	
H11A	0.6957	0.6868	0.9016	0.020*	
C12	0.8105 (4)	1.0173 (9)	0.7811 (3)	0.0188 (8)	
H1O3	0.721 (5)	1.068 (16)	0.582 (4)	0.025 (16)*	
H1N1	1.027 (5)	−0.101 (9)	0.256 (4)	0.023 (12)*	
H1N2	1.000 (6)	−0.248 (12)	0.092 (4)	0.039 (15)*	
H2N2	0.898 (4)	−0.093 (8)	0.013 (4)	0.021 (11)*	

### Atomic displacement parameters (Å^2^)

**Table:**

	*U*^11^	*U*^22^	*U*^33^	*U*^12^	*U*^13^	*U*^23^
Br1	0.02863 (18)	0.02674 (19)	0.01847 (16)	0.0061 (3)	−0.00223 (12)	−0.0056 (2)
Cl1	0.0287 (5)	0.0241 (5)	0.0232 (5)	−0.0076 (4)	−0.0007 (4)	0.0025 (4)
O1	0.0301 (12)	0.0291 (17)	0.0151 (11)	−0.0079 (18)	−0.0073 (9)	0.0053 (16)
O2	0.0256 (14)	0.0202 (14)	0.0158 (13)	−0.0045 (12)	−0.0028 (11)	0.0015 (11)
O3	0.0306 (18)	0.0268 (18)	0.0141 (14)	−0.0079 (15)	−0.0061 (12)	0.0035 (12)
N1	0.0225 (15)	0.020 (2)	0.0148 (13)	0.0051 (15)	−0.0014 (11)	−0.0013 (13)
N2	0.0232 (18)	0.027 (2)	0.0128 (16)	0.0059 (15)	−0.0048 (13)	−0.0038 (14)
C1	0.0196 (15)	0.0188 (19)	0.0147 (14)	0.001 (2)	0.0000 (11)	0.005 (2)
C2	0.023 (2)	0.021 (2)	0.0151 (17)	0.0002 (16)	−0.0060 (15)	0.0048 (15)
C3	0.0193 (19)	0.019 (2)	0.0204 (19)	0.0038 (16)	−0.0021 (15)	0.0031 (15)
C4	0.0206 (19)	0.022 (2)	0.0136 (17)	0.0026 (16)	0.0004 (14)	−0.0021 (15)
C5	0.027 (2)	0.022 (2)	0.0129 (17)	−0.0013 (17)	−0.0025 (15)	0.0010 (15)
C6	0.0192 (18)	0.0173 (19)	0.0119 (16)	0.0013 (15)	−0.0006 (13)	−0.0022 (14)
C7	0.0157 (17)	0.021 (2)	0.0158 (17)	−0.0011 (16)	−0.0032 (13)	−0.0027 (16)
C8	0.0209 (18)	0.027 (2)	0.0188 (18)	−0.0023 (16)	−0.0037 (15)	−0.0038 (15)
C9	0.0183 (18)	0.023 (2)	0.0218 (19)	−0.0046 (15)	−0.0005 (15)	−0.0051 (15)
C10	0.0198 (17)	0.016 (3)	0.0199 (17)	0.0029 (14)	0.0022 (14)	−0.0014 (14)
C11	0.0192 (18)	0.018 (2)	0.0130 (16)	−0.0001 (15)	−0.0019 (14)	−0.0011 (14)
C12	0.0198 (16)	0.019 (2)	0.0171 (15)	0.0002 (17)	−0.0022 (12)	0.0006 (17)

### Geometric parameters (Å, º)

**Table:**

Br1—C4	1.893 (4)	C3—C4	1.399 (5)
Cl1—C10	1.754 (4)	C3—H3A	0.9500
O1—C12	1.252 (4)	C4—C5	1.350 (5)
O2—C12	1.284 (5)	C5—H5A	0.9500
O3—C7	1.359 (5)	C6—C7	1.396 (5)
O3—H1O3	0.77 (8)	C6—C11	1.398 (5)
N1—C1	1.346 (4)	C6—C12	1.483 (6)
N1—C5	1.357 (5)	C7—C8	1.393 (5)
N1—H1N1	0.86 (5)	C8—C9	1.382 (6)
N2—C1	1.321 (6)	C8—H8A	0.9500
N2—H1N2	0.96 (6)	C9—C10	1.389 (5)
N2—H2N2	0.88 (5)	C9—H9A	0.9500
C1—C2	1.412 (6)	C10—C11	1.375 (5)
C2—C3	1.372 (6)	C11—H11A	0.9500
C2—H2A	0.9500		
			
C7—O3—H1O3	123 (4)	C7—C6—C11	118.7 (4)
C1—N1—C5	122.5 (4)	C7—C6—C12	122.1 (3)
C1—N1—H1N1	119 (3)	C11—C6—C12	119.2 (3)
C5—N1—H1N1	118 (3)	O3—C7—C8	117.7 (3)
C1—N2—H1N2	120 (3)	O3—C7—C6	121.9 (4)
C1—N2—H2N2	117 (3)	C8—C7—C6	120.3 (4)
H1N2—N2—H2N2	122 (4)	C9—C8—C7	120.6 (4)
N2—C1—N1	118.4 (4)	C9—C8—H8A	119.7
N2—C1—C2	123.1 (3)	C7—C8—H8A	119.7
N1—C1—C2	118.5 (4)	C8—C9—C10	118.7 (4)
C3—C2—C1	119.3 (4)	C8—C9—H9A	120.6
C3—C2—H2A	120.4	C10—C9—H9A	120.6
C1—C2—H2A	120.4	C11—C10—C9	121.5 (4)
C2—C3—C4	120.0 (4)	C11—C10—Cl1	119.6 (3)
C2—C3—H3A	120.0	C9—C10—Cl1	118.9 (3)
C4—C3—H3A	120.0	C10—C11—C6	120.1 (3)
C5—C4—C3	119.6 (4)	C10—C11—H11A	120.0
C5—C4—Br1	120.3 (3)	C6—C11—H11A	120.0
C3—C4—Br1	120.1 (3)	O1—C12—O2	122.9 (4)
C4—C5—N1	120.2 (4)	O1—C12—C6	119.7 (4)
C4—C5—H5A	119.9	O2—C12—C6	117.4 (3)
N1—C5—H5A	119.9		
			
C5—N1—C1—N2	179.6 (4)	O3—C7—C8—C9	177.9 (4)
C5—N1—C1—C2	0.6 (6)	C6—C7—C8—C9	0.0 (6)
N2—C1—C2—C3	−179.6 (4)	C7—C8—C9—C10	0.9 (6)
N1—C1—C2—C3	−0.5 (6)	C8—C9—C10—C11	−0.9 (6)
C1—C2—C3—C4	0.7 (6)	C8—C9—C10—Cl1	178.9 (3)
C2—C3—C4—C5	−1.0 (6)	C9—C10—C11—C6	0.1 (5)
C2—C3—C4—Br1	179.8 (3)	Cl1—C10—C11—C6	−179.7 (3)
C3—C4—C5—N1	1.0 (6)	C7—C6—C11—C10	0.8 (5)
Br1—C4—C5—N1	−179.7 (3)	C12—C6—C11—C10	−179.4 (3)
C1—N1—C5—C4	−0.8 (6)	C7—C6—C12—O1	−178.9 (4)
C11—C6—C7—O3	−178.6 (3)	C11—C6—C12—O1	1.3 (6)
C12—C6—C7—O3	1.6 (6)	C7—C6—C12—O2	1.1 (5)
C11—C6—C7—C8	−0.8 (6)	C11—C6—C12—O2	−178.7 (3)
C12—C6—C7—C8	179.4 (3)		

### Hydrogen-bond geometry (Å, º)

**Table:**

*D*—H···*A*	*D*—H	H···*A*	*D*···*A*	*D*—H···*A*
O3—H1*O*3···O2	0.77 (8)	2.02 (5)	2.553 (4)	127 (6)
N1—H1*N*1···O2^i^	0.86 (5)	1.82 (5)	2.666 (4)	172 (4)
N2—H1*N*2···O1^i^	0.96 (6)	1.81 (6)	2.770 (5)	175 (4)
N2—H2*N*2···O1^ii^	0.88 (5)	1.95 (5)	2.799 (5)	164 (3)
C8—H8*A*···O3^iii^	0.95	2.53	3.410 (5)	154

Symmetry codes: (i) −*x*+2, *y*−3/2, −*z*+1; (ii) *x*, *y*−1, *z*−1; (iii) −*x*+1, *y*−1/2, −*z*+1.
